# Bridging care in brain injury rehabilitation: A reflexive thematic analysis of staff perspectives on the role of peer support

**DOI:** 10.1111/bjhp.70049

**Published:** 2026-01-18

**Authors:** Stephen Dunne, Adele Simpson, Jaden Allan

**Affiliations:** ^1^ School of Psychology, Faculty of Health and Wellbeing, Northumbria University Newcastle‐upon‐Tyne UK; ^2^ Northumberland Head Injuries Service (NHIS), Cumbria, Northumberland, Tyne and Wear NHS Foundation Trust Morpeth UK; ^3^ School of Healthcare and Nursing Sciences, Faculty of Health and Wellbeing, Northumbria University Newcastle‐upon‐Tyne UK

**Keywords:** brain injury, living experience, neurorehabilitation, peer support, qualitative research

## Abstract

**Objectives:**

Peer support roles are well established in mental health services, yet their integration into neurorehabilitation remains limited. This study offers novel insight into how staff perceive the opportunities and challenges of introducing a peer support worker role into a community brain injury service.

**Design:**

Using qualitative interviews and focus groups with clinical and support staff, we explored understandings of the role, its perceived value and contextual barriers to implementation.

**Methods:**

Participants included eight staff members (100% female; Mage = 49.88). Data were analysed using reflexive thematic analysis. Rigour was ensured through pre‐registration, triangulated coding discussions and reflexive analytic practices.

**Results:**

Three themes were constructed: the perfect candidate, context for success and connecting care. Findings highlighted the unique value of lived experience in fostering trust, hope and engagement, while also revealing neuro‐specific challenges relating to cognition, behaviour and role boundaries. Staff emphasized the need for tailored recruitment, training and supervision frameworks distinct from existing mental health models.

**Conclusions:**

The study is significant in being the first to systematically examine the adaptation of peer support to neurorehabilitation, offering evidence to guide service development and policy. Collectively, the findings underscore both the transformative potential and the structural requirements of embedding peer support within neurorehabilitation contexts.

**Statement of Contribution**

**What is already known on this subject?**

- Peer support is well established in mental health services.
- Evidence on peer support in neurorehabilitation, especially brain injury, is minimal.
- Challenges unique to neurological conditions may influence role integration and effectiveness.

**What does this study add?**

- Shows staff value living experience in fostering trust and hope.
- Identifies neuro‐specific challenges requiring tailored support.
- Provides first evidence to guide peer support adaptation in neurorehabilitation.

## INTRODUCTION

Traumatic brain injury (TBI), defined as an injury to the brain caused by trauma to the head (Headway, 2016; NICE, 2023), affects 10 million people annually worldwide and is an increasing cause of death and disability (Hyder et al., 2007; Maas et al., 2017). The scale of TBI in the United Kingdom is similarly substantial, with over 160,000 people attending hospital every year with head injuries (Hassan et al., 2013) and approximately 1.3 million people living with disabilities resulting from head injury (Centre for Mental Health, 2016).

Following TBI, individuals may be left with impairments of varying severity, including physical, cognitive, emotional and behavioural problems (Norlander et al., 2016). These issues can significantly impact self‐esteem and identity (Salter et al., 2008), hindering social connections and engagement (Carod‐Artal, 2012). Several studies highlight that brain injury is associated with a decrease in the quantity or quality of friendships, social contacts and meaningful relationships (Dijkers, 2004; Douglas, 2020). The resulting loneliness and social isolation are widely reported as long‐term consequences of living with acquired brain injury (Kumar et al., 2020). Even after adapting to physical impairments, many survivors continue to face difficulties in maintaining social contacts and significant relationships (Tomberg et al., 2005). Consequently, TBI can have profound and enduring effects on wellbeing and quality of life (Haley et al., 2011; Hawthorne et al., 2009), often manifesting as chronic mental health conditions such as anxiety and depression (Fleminger et al., 2003; Proctor & Best, 2019).

Given these challenges, there is increasing recognition of the need for psychosocial interventions that complement medical and rehabilitation care. One such approach is peer support, sometimes described as ‘peer mentoring’ or ‘peer coaching’, in which individuals draw on their own health‐related experiences to support others facing similar circumstances (Levy et al., 2019; Wasilewski et al., 2023, 2025). Peer support is increasingly recognized as integral to the rehabilitation process, not only addressing informational and emotional needs but also fostering hope, empowerment and social connection (Badger & Royse, 2010a, 2010b; Jones & Gassaway, 2016; Vincent et al., 2015; Zatzick et al., 2018). Peers can act as a crucial bridge between patients, their families and healthcare professionals (Davidson et al., 2012; Leamy et al., 2011; Slade et al., 2014). Several psychological theories help explain why peer support may be particularly impactful for brain injury survivors. Peer support theory emphasizes shared responsibility, mutual agreement on what is helpful, and relational equality (Mead et al., 2021), while social support frameworks (House et al., 1988) highlight the emotional, informational and appraisal‐based functions peers are uniquely positioned to provide. Social learning theory (Bandura, 1977) further suggests that peer workers can act as credible role models whose visible recovery enhances survivors’ self‐efficacy and belief in achievable progress. At the same time, peer‐to‐peer support can present challenges, including differing perspectives on illness and recovery, variations in relational fit and reluctance to disclose personal experiences (Davidson et al., 2012; Wasilewski et al., 2025).

Evidence from mental health settings suggests that peer support can reduce hospitalizations, decrease reliance on outpatient services, mitigate social isolation and improve community integration (Mental Health Foundation, 2019). Emerging research specific to TBI populations indicates that peer support may alleviate post‐injury distress, enhance well‐being and build resilience against mood disorders (Hibbard et al., 2002; Reichmann & Bartman, 2018). The personalized nature of peer support, offering emotional reassurance, practical advice and validation, may help TBI survivors navigate identity changes, adapt to new limitations and re‐engage with society (Badger & Royse, 2010a, 2010b; Dunne et al., 2023; Yang et al., 2022).

However, despite its potential, the implementation of peer support roles within neurorehabilitation remains under‐researched, particularly in UK contexts. Existing literature has tended to focus on the neurological effects of TBI and the benefits of peer support, with less attention to the practical, organizational and cultural factors that influence successful integration into healthcare teams (Levy et al., 2019). Studies have highlighted the importance of staff ‘buy‐in’ for the sustainability of peer support programmes (Bradford et al., 2013; Wasilewski et al., 2023), as well as the need to manage potential risks, such as mismatched recovery trajectories or emotional strain for peer supporters (Salas et al., 2022; Wasilewski et al., 2023).

Northumberland Head Injuries Services, in the Northeast of England, provide a rare example of employing a paid peer supporter to deliver structured support to TBI clients as part of routine service provision. The peer supporter completes a structured induction and training programme, including mandatory service induction (safeguarding, information governance), a ‘Brain Injury Awareness’ session led by the Service Lead, and an induction at a specialist neurorehabilitation hospital to broaden understanding of TBI presentations and services. This is followed by several weeks of shadowing the multidisciplinary team to familiarize themselves with clinical sessions and team processes. Ongoing supervision is provided by both the neurorehabilitation team and the peer support service, addressing client‐specific and general peer support needs. This creates a valuable opportunity to explore the perceived benefits, challenges and contextual factors influencing the integration of such roles into neurorehabilitation.

The present qualitative service evaluation aimed to: (1) explore staff expectations of a peer support worker role; (2) identify perceived barriers to hiring and sustaining such roles in neurorehabilitation; and (3) develop evidence‐informed recommendations for effective implementation. By addressing these aims, the study seeks to contribute to the limited evidence base on peer support roles in TBI services and provide practical insights for wider adoption in similar contexts.

## MATERIALS AND METHODS

The study design and analysis plan were preregistered prior to data collection to enhance transparency and methodological rigour. The pre‐registration is publicly available at https://osf.io/zfua4/. All materials associated with the study are publicly available on the project page: https://osf.io/xr6vm (Dunne et al. 2026).

### Ethical approval

Completion of the National Health Service Health Research Authority decision‐making questionnaire confirmed that formal ethical approval was not required for the study. The NHS Trust Research Office peer reviewed and approved the project proposal (SER‐23‐211) and verified that no further local approvals were required for this evaluation to take place.

While not requiring formal ethical approval, we sought to maintain high ethical standards in this project. Informed consent was received from all interviewees for interview participation and the sharing of de‐identified data. All documents and project materials were reviewed by the authors' University Ethics Committee in the UK (Ref: 5805).

### Design

We adopted a constructivist paradigm with an interpretive epistemology (Crotty, 1998; Denzin & Lincoln, 2018), chosen for its focus on the co‐construction of meaning between researchers and participants. This was appropriate for exploring how staff understand and negotiate the role of peer support in neurorehabilitation, where perspectives are shaped by both individual experience and shared organizational context.

A combination of focus groups and semi‐structured interviews was used to balance collective dialogue with opportunities for more personal reflection. This dual‐method design enabled a richer account of staff perspectives than would have been possible through either method alone.

An inductive analytic approach supported the prioritization of participants' lived experiences and avoided imposing pre‐existing theoretical frameworks. While findings are inevitably context‐dependent and co‐produced rather than objective truths, this design ensures that the insights are grounded in the realities of service delivery. Transferability is therefore limited, but the study offers nuanced, practice‐relevant contributions. The study was reported in line with SRQR standards (O'Brien et al., 2014) to enhance transparency and consistency.

### Recruitment and participants

Participants were recruited through the NHIS. After an initial meeting to explain the project to staff members, participants emailed the lead researcher to take part. To be eligible to participate, participants had to be actively working for the NHIS. The research team made sure to recruit participants with a range of job titles and experience from within the service to provide a holistic voice. In total, 8 staff members agreed to take part (8 females; 37–66 years old; M_age_ = 49.88, SD_age_ = 8.85) agreed to participate in the study. Participant demographics can be found in Table 1.

**TABLE 1:** Participant characteristics.

Participant #	Activity	Age	Gender	Job role	Years in role
1	Interview A	37	F	Speech and Language Therapist	8
2	Dyadic Interview A	55	F	Physiotherapist	14
3	Dyadic Interview A	52	F	Apprentice Occupational Therapist	3
4	Focus Group B	50	F	Community Practitioner	5
5	Focus Group B	52	F	Administration Officer	13
6	Focus Group B	37	F	Occupational Therapist	7
7	Interview B	50	F	Occupational Therapist	24
8	Interview C	66	F	Assistant Community Practitioner	24

Participants who volunteered for the study were sent an information sheet and consent form electronically prior to interview, allowing them time to read the study information and decide whether to participate. During participation, this practice was repeated, with the researcher reading the information sheet and consent form aloud, ensuring that participants had comprehended each element prior to participation. Of the eight participants, three took part in individual semi‐structured interviews with the lead author, two participated in a dyadic interview, and three joined a focus group. The mode of participation was determined by individual preference, availability and service pressures to ensure minimal disruption to clinical duties. This flexible approach allowed for efficient data collection while maximizing engagement from staff within the neurorehabilitation service.

### Procedure

Participants were invited to take part in either a one‐to‐one semi‐structured interview or a focus group, depending on personal preference and scheduling considerations. An online participant information sheet was provided in advance, and participants were allocated a unique code number for file storage purposes. Consent was obtained electronically prior to the session and reconfirmed verbally before recording commenced, using a standardized introduction script.

Interviews were conducted by SD alone while focus groups were facilitated by SD and JA jointly and followed an interview schedule of 13 open‐ended questions, with additional probes used as appropriate to explore emerging topics in depth. Sessions were anticipated to last between 60 and 90 min and were audio‐recorded with permission. Participants were reminded of their right to pause, stop or withdraw from the session at any time without consequence. Upon completion, participants were emailed a debrief information sheet containing details of relevant support services.

Audio recordings were transcribed verbatim following the orthographic transcription system described by Clarke and Braun (2013). Emotional expressions and common non‐verbal utterances were denoted in double parentheses (()), and identifying information was removed during first‐draft transcription, with pseudonyms allocated according to participant choice. All electronic data were encrypted and stored securely on a password‐protected server. Data collection continued until the dataset was judged sufficiently rich to enable robust theme generation.

### Data processing and analysis

Recordings were transcribed and checked for accuracy against the audio files before being imported into Microsoft Word for coding and organisation. Data were analysed using Reflexive Thematic Analysis (RTA) as outlined by Braun and Clarke (2006, 2022), adopting an inductive, data‐driven approach.

Analysis was led by SD in conjunction with AS and JA and followed the six phases of RTA: (1) familiarization with the data; (2) coding; (3) generating initial themes; (4) developing and reviewing themes; (5) refining, defining and naming themes; and (6) producing the report. Familiarization was facilitated by SD and JA's dual role in data collection and transcription, with repeated reading and critical reflection on each transcript. Initial coding was primarily semantic, capturing participants' explicit accounts before moving to interpretive analysis to identify underlying meanings and shared patterns across the dataset. After every two transcripts were coded, all authors held regular triangulation meetings to compare interpretations, refine codes and ensure consistency in the analytical approach.

Theme generation was iterative and reflexive, with candidate themes reviewed and refined to ensure analytic coherence and relevance to the research aims. Analysis was guided by the 15‐point Thematic Analysis checklist (Clarke & Braun, 2013) and the ‘Twenty Questions to Guide Assessment of TA Research Quality’ (Braun & Clarke, 2021) to maintain methodological rigour.

### Researcher reflexivity

Three researchers contributed to the study, each bringing perspectives that may have influenced the research process. SD, a mixed‐methods researcher specializing in stroke and brain injury, prioritized capturing staff perspectives to support applied service improvements, while maintaining an outsider view to reduce organizational preconceptions. JA, an Assistant Professor with clinical nursing experience and expertise in peer support, provided a practice‐informed lens, emphasizing inclusivity and co‐production. AS, a Principal Clinical Psychologist within the Northumberland Head Injuries Service, offered insider knowledge of the clinical context, enhancing interpretation. Reflexive discussions among the team were maintained throughout analysis to surface and address differing perspectives, supporting transparency and rigour in interpreting staff experiences.

### Techniques to enhance rigour

Multiple strategies were employed to ensure the credibility, transparency and trustworthiness of the analysis. A reflective diary was maintained by all authors throughout the study to create an audit trail and capture reflexive engagement with the data, including emotional responses and interpretive decisions. This practice aligns with the reflexive principles of RTA and the study's social constructivist orientation, recognizing the research team's individual expertise as an asset to interpretation rather than a source of bias (Braun & Clarke, 2019; Byrne, 2022).

To enhance transparency, the project was preregistered on the Open Science Framework (OSF), and the analytic process was documented in detail and available on the project page. Transcriptions were produced to a high level of detail, checked against the recordings for accuracy and anonymized during first‐draft transcription. Coding and theme development were revisited iteratively, with provisional themes critically appraised against the data set and refined to ensure analytic depth.

Rigour was further supported by adherence to the 15‐point Thematic Analysis checklist (Clarke & Braun, 2013) and the 20‐point quality questions (Braun & Clarke, 2021). The regular triangulation meetings between all authors after coding every two transcripts also served to challenge assumptions, verify interpretations and reduce individual researcher bias.

## RESULTS

Three clear themes were constructed from the analysis: the perfect candidate; context for success; connecting care.

### The perfect candidate

Staff had clear expectations of what an ideal peer support worker should embody, with a particular emphasis on the balance between living experiences and the ability to uphold professional responsibilities. Participants' living experiences were always seen as central to the role's value.

I see it as someone who's had a lived experience … Because they have got a unique insight… I can talk to someone about how to manage their fatigue but I haven't suffered the fatigue the same as someone with a head injury, so how can I tell them, “if you do x, y and z, it's going to work”? … Having someone else that's gone through it and just has that… Gets it. (P7)

It can be a bit of a challenge. As much as I try and put myself in their shoes and… And try and imagine how they might be feeling; I don't have that experience. …it would be more powerful and perhaps more reassuring and more of a confidence builder to have somebody there who's been there, done that, to provide their experience. (P1)

While all participants spoke of the importance of living experiences and the benefit it will provide not only for families and the service, they also expressed concerns about how this living experience of brain injury might impact their ability to maintain professional requirements. Specifically, staff questioned whether individuals with brain injuries could maintain professional boundaries, confidentiality and adherence to organizational expectations. At the same time, some concerns reflected the need for formalized training, supervision and governance structures to support peer workers in meeting these standards. This suggests that anxieties were not solely about the peer workers' lived experience per se, but also about ensuring appropriate system‐level supports were in place to enable safe and professional practice.

Trust training and it's drummed into us, erm, it's [required] by our professional standards that we need to maintain. Erm, our codes of conduct in order to practice. They will have potentially no professional background. Erm, and therefore won't work to those standards. Maybe they've never appreciated what is confidentiality and what you can share and what you can't and, er, if they're going from client to client, you don't want them saying to this client about that client. (P3)

I suppose we keep everything confidential [professional requirement] all the time, and everything's confidential [professional requirement], that you don't share with patients, and then suddenly we have a patient who may well have been in our service before. It might not be, who suddenly has access to RIO [electronic patient record system used within the NHS], who hasn't gone through any formal training around information governance, around… You know, you're just brought up with it within the NHS, when you're within a profession (P3)

My concerns initially were …around the governance…if I'm in a meeting and I've got to say, “oh, these medical…” Or there might be some safeguarding [sensitive medical and client information]. … that's a little bit uncomfortable and there's somebody there that was a patient. I think, it's just an adjustment, isn't it? It's new. I've not done it before. (P4)

There were also concerns about whether the impairments a peer supporter is living with would affect role performance. Some participants worried about cognitive or emotional challenges influencing reliability or communication.

I suppose I'm a little bit apprehensive because obviously this person has had a head injury; they're going to have needs and stuff, erm, of their own, that… Kind of, we expect a certain standard from each other, that we might not be able to get with a peer support worker, because they've got whatever cognitive issues, or… there is those kind of concerns of maybe the wrong thing being said. (P2)

To find the right person that will fit in with the team, that is able to provide the level of professional, erm, professionality that we need, but also have enough lived experience of a significant head injury to be able to give that lived experience, but not be so impaired that they can't actually deliver on that. It's a really fine balance and one of my concerns… (P5)

This concept highlights a fundamental tension at the heart of staff perceptions: the desire to value and incorporate living experiences while ensuring that the peer support worker can meet the professional demands of the role. Participants often spoke about the need to find the ‘right person’, someone whose own experience of brain injury was authentic and relatable, yet whose impairments did not significantly impact their ability to perform the role effectively. There was a sense that staff were seeking a ‘magic individual’ [P4] with a range of qualities; enough living experience to offer genuine empathy and insight, but minimal functional limitations that might affect reliability, communication or boundary‐setting.

### Context for success

The success of a peer support worker was seen as highly dependent on the surrounding team and the broader NHS system. Staff repeatedly emphasized that the success of the role would not hinge on the individual alone, but rather on the strength of the team surrounding them and the wider NHS system in which they would be embedded. While system‐level challenges were acknowledged, it was the immediate team environment at Northumberland Head Injury Services that emerged as a particularly strong facilitator, seen by staff as the foundation on which the success of the peer support role would be built.

I think we're a very good team at accommodating everyone's needs. (P7)

The team was unanimously described as a key strength. Participants spoke with pride about the supportive, communicative and inclusive culture of their team, which they believed would provide a strong foundation for integrating a peer support worker and facilitate the effectiveness of the role. Although the service was acknowledged to be in a period of transition, staff remained confident that their team's shared values, openness to new roles and commitment to patient‐centred care would act as a major facilitator. There was a clear sense that the team would not only welcome a peer support worker but also actively support their development, offer guidance and help them navigate the complexities of the role. This culture of support was seen as essential to ensuring the peer support worker felt empowered, valued and part of the wider clinical team.

This team, particularly, is very good at trying to get people to be as independent as possible. (P7)

It is a welcoming place to be… I suppose we're just fortunate with the personalities, but I think that the culture is there, that we share and that we support. (P2)

While the team itself was viewed as a strong facilitator, staff were clear that wider NHS structures and mandatory training requirements could present significant barriers to the successful implementation of a peer support worker role. A key concern was the rigidity of mandatory NHS training requirements, which staff felt could pose unnecessary challenges for individuals post‐brain injury.

…hiring someone with a brain injury to a role is a challenge in itself, because there's a lot that they're going to be potentially dealing with themselves and will continue to deal with, you know? Erm, insight, fatigue, a range of different issues that still persist. Brain injury is most often a constant process. Is it a double‐edged sword then of them to then deal with potential NHS organisational aspects as well? (P7)

I think the environmental factors, which are, I suppose the culture we're in at the minute, that we don't have a permanent base, this is temporary, we're split across the two sides of the department and we are in individual offices, erm… Whereas I think, erm, what we would be wanting to do – … is that peer support worker would be in with the community practitioners… (P8)

There was particular unease around the peer support training currently offered within the NHS, which staff had recently undertaken. They described this training as being heavily focused on mental health contexts, with limited consideration of the specific needs and realities of brain injury. As a result, staff felt the training not only lacked relevance but also risked misrepresenting what peer support could and should look like in neurorehabilitation settings. Rather than feeling better equipped, some staff reported that the training created confusion and unhelpful preconceptions about the role, highlighting a clear need for more tailored, condition‐specific training that reflects the unique challenges and opportunities within brain injury services.

I mean, you know, we've had in the past, patients that have presented… But they're perhaps, you know, a bit disinhibited … and you wouldn't necessarily know that at an interview, so those… And I didn't feel that the peer support… I don't know what to call them; the group that were implementing this, erm, I don't know that they fully understood all that, which they would need to. (P8)

They're the ones that guided us and they were the ones that we had the meetings with initially to talk about it. Erm, and it was… And they obviously had mental health problems themselves, you know, in the past and sort of… I didn't feel totally grasped that. And why would you? You know, that's not a criticism. It's like, why would you understand that. Erm, and I felt that …, it was like, “oh, we'll just do this and we'll just do that” and I'm like, oh, I'm not sure about that. (P5)

This gap in bespoke training was seen as a critical oversight. Staff expressed concern that without targeted, neurorehabilitation‐specific training, peer support workers may be underprepared for the realities of the role, and teams may lack the guidance needed to integrate them effectively. Without this tailored support, there was a risk that the role could be misunderstood, underutilized or implemented in ways that failed to reflect the unique needs of brain injury services.

I know they've got lived experience of traumatic brain injury, but just that awareness of the breadth of presentations, erm, which they may have some through the education they've already had, erm, but yes, I'd want to be providing them with a bit of general education about brain injury generally and then probably also within individual disciplines. (P1)

However, that peer support worker is going to need to be supported themselves. Especially initially, I suppose. Erm, so that's where I see the perhaps… I don't want to say ‘drain’ on the service, but something that needs to be…[time invested in]. (P4)

### Connecting care

One of the most widely acknowledged benefits of a peer support worker was their potential to act as a crucial intermediary between clients, families and the healthcare team.

How valuable would it be to have somebody with lived experience sitting there and saying, “I was where you were a year ago, ten years ago” whatever it is. Erm, “I faced these challenges. I felt just how you're reporting you feel, erm, but I put this, this and this in place. I pushed through with the rehab. I had ups and downs. Erm, and now I'm here”. Erm, and likewise with families, I suppose, being for families to be able to see that potentially there's light at the end of the tunnel. Yeah, there's other people that have gone through things. (P1)

The family member may ask the questions that they maybe were scared to ask us, or, erm… So I think that the client's family will be supported by it and I think it'll facilitate an engagement of the client as well. (P3)

Participants frequently described the peer support worker role as a vital connector within the care system. The peer supporter was seen as someone who could bridge gaps between clients, families and clinical teams in ways that traditional NHS roles often could not. Staff highlighted that the unique value of the peer support worker lay not only in their living experience, but also in their ability to build trust and rapport with clients on a different level. Unlike clinicians, who are often bound by strict professional boundaries and system constraints, peer support workers were seen as more accessible and relatable, able to engage with clients in a more informal and emotionally resonant way.

We have to manage boundaries as clinicians all the time. Some clients might be a bit more familiar, but as a therapist It's relatively easy to have your strict boundaries and you maintain those. I just… Perhaps… I don't know. It feels like there's a potential for it to be a little bit less clear with a peer support worker. (P1)

This positioning allowed them to act as a bridge, not just between people and services, but between personal experience and professional care. Staff described this role as enhancing continuity and cohesion in service delivery, helping clients and families feel more connected, understood and supported throughout their neurorehabilitation journey. Many staff saw the peer support worker as a ‘lynchpin’ in strengthening relationships across the system, facilitating more joined‐up working, improving communication and ultimately helping to create a more compassionate and responsive care environment.

It's down to engagement and them maybe sharing with the peer support worker things they might not share with us, ‘cos it might seem trivial’. (P2)

The success would be for the peer support worker and then positive feedback from the clients. You know if they say, “I know you said it, but it made so much sense when it came from somebody who's actually had a head injury”. (P5)

There was a genuine sense of excitement among staff about the potential for the peer support worker role to enhance client engagement and motivation, with many viewing it as a powerful tool to inspire hope and promote more active participation in rehabilitation.

It can be a springboard for somebody. It could be a way back into employment, where they hadn't… That would be really nice. To find somebody who hasn't been able to find a role for whatever reason and, you know, having been given this opportunity, it opens the door to other things. (P5)

You know, to say to somebody, … “you've been given this opportunity… You've grown in confidence; you've grown in knowledge and skills…” They see a role that actually previously would have been unachievable, or they felt unachievable for themselves, but having had this experience with us, it's given the confidence to be able to go for that role. (P4)

Despite the enthusiasm surrounding the potential of the peer support worker to improve engagement, some staff also expressed caution about the unintended consequences of such positivity. There were concerns that clients might compare their own progress to that of the peer support worker, especially if the peer had made significant gains in their recovery. This raised important questions about how to manage expectations, both for clients and within the service, around what recovery can realistically look like. While the peer's story could offer hope, it also had the potential to create pressure or disappointment if clients felt they were not progressing at a similar pace or achieving comparable outcomes. Staff reflected on the need to strike a balance between inspiration and realism and highlighted the importance of ongoing support and communication to ensure that the role empowered rather than inadvertently discouraged clients.

I think it's really key that… You get someone that's able to manage the demands of that role. But in doing so, it's whether some of the clients that the individual might be supporting, whether they'll be able to see themselves in that individual. (P1)

My only other concern, is… You know we recently had a student and on her first week, she was like hearing all these stories, talking to all these patients and she was like, “how do you deal with all these really like, traumatic stories?” And I was like… And it's probably because I've done the job so long that I'm just like… You what? But it got me thinking, if had a peer supporter, you don't want anything to be triggered for them. (P7)

I think you'd have to make sure that you say to the peer supporter, “look”. Everyone is different and everyone is going to recover at their own rate. And if you can accept that and don't give them false promises… You know? Don't say, ‘well look – I wasn't walking six months ago. Now look at me’, because that's not going to help them. But what will help them is knowing that actually it is hard, and tiredness is knackering, but just do little by little, and coping with the change… (P8)

Despite these concerns, the overall sentiment among staff remained overwhelmingly positive. Most participants spoke passionately about the value a peer support worker could bring, not only for clients, but particularly for families, who often need additional guidance and reassurance. The role was consistently framed as an incredible resource with the potential to strengthen service delivery, enhance the rehabilitation experience, and ultimately contribute to a more holistic, compassionate and effective support system for those navigating life after brain injury.

**FIGURE 1:**
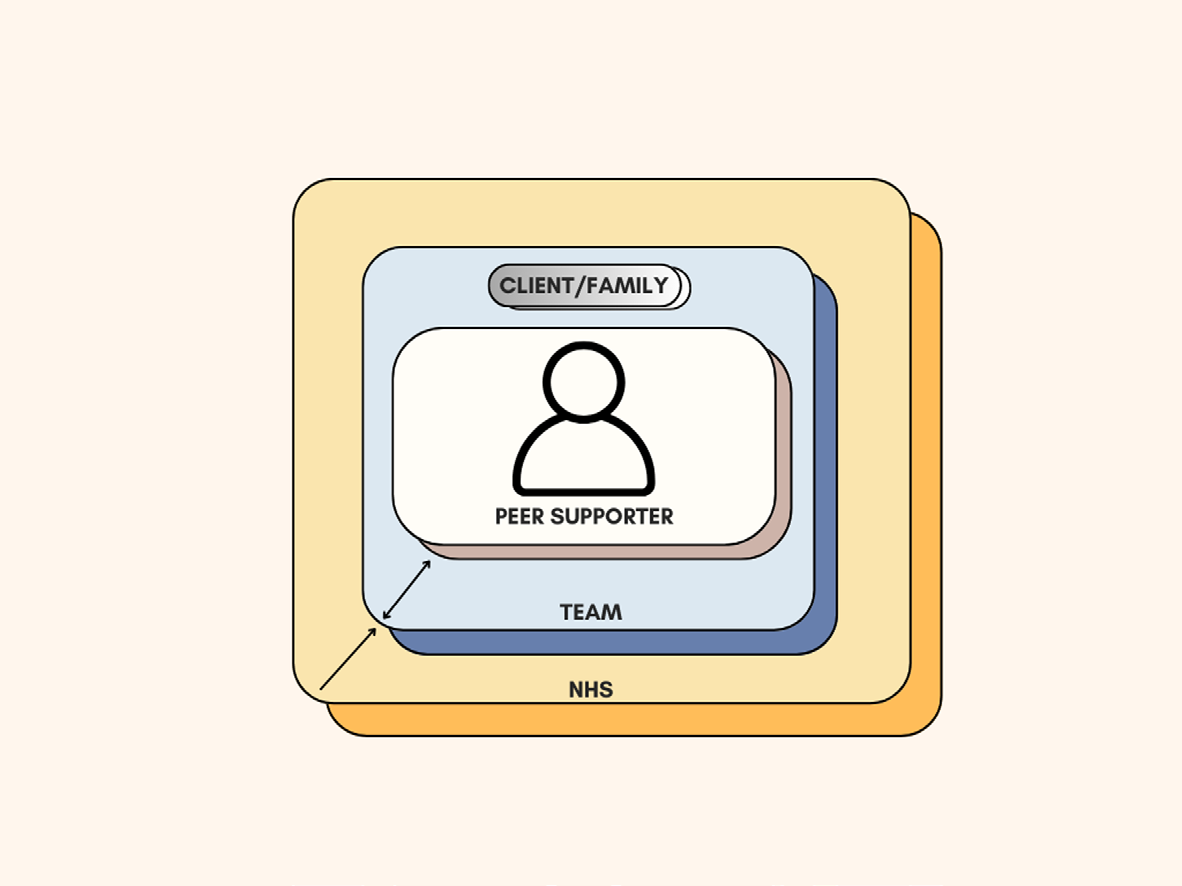
Visualization of the peer support worker's role within neurorehabilitation services, illustrating their central position as a role model and bridge for clients and families, integrated within the clinical team, and shaped by wider NHS system enablers and constraints.

To complement the themes presented, Figure 1 offers a visualiation of the core discussions held with staff members, highlighting the interconnections between expectations, systemic factors and the envisioned role of a peer support worker.

At the centre of this visualiation sits, the peer support worker, viewed by participants as a lynchpin within the service. Staff described the role as multifaceted. Not only was the peer supporter a visible symbol of progress for clients, a relatable role model, and a bridge between families and clinical teams, but also a source of hope and reassurance throughout rehabilitation. Surrounding this central figure is the team itself, within which the peer support worker is envisioned to be fully integrated, not as a patient, but as a valued member of staff. However, discussions also revealed a team in transition, navigating differing understandings of the peer support worker's role, responsibilities and positioning within existing team structures. Encasing both of these elements is the broader NHS system, which staff described as both enabling and constraining. Concerns were raised about the appropriateness of current NHS peer support training, largely designed for mental health contexts, alongside practical barriers such as workloading, limited resources and the need for additional supervision. This layered visualization reflects the complex ecosystem in which the peer support worker is expected to operate, highlighting the dynamic interplay between individual, team and systemic factors that will ultimately shape the success of the role.

## DISCUSSION

This study aimed to explore staff expectations of a peer supporter role in neurorehabilitation, identify perceived barriers to implementation and provide recommendations for successful integration. Through qualitative interviews with staff at a brain injury service, three interrelated themes were constructed: the perfect candidate, context for success and connecting care. Together, these themes reflect a clear recognition of the peer supporter's potential to enhance service delivery and support recovery, while also surfacing the structural, cultural and ethical tensions that must be addressed for successful implementation.

While peer support roles are well established within mental health services (see Repper & Carter, 2011 for a review), their translation into neurorehabilitation is limited and requires careful consideration of context‐specific factors. Our findings suggest that staff perceive the peer support worker as a highly novel and pivotal addition to brain injury services, acting as a ‘lynchpin’ in fostering trust, hope and continuity of care between clients, families and the clinical team. Consistent with literature highlighting the value of living experiences in peer support (Cooper et al., 2024; Smit et al., 2023), participants emphasized that authenticity grounded in personal experience of brain injury is central to the perceived effectiveness of the role. However, staff also recognized that neuro‐specific cognitive, emotional and behavioural sequelae introduce challenges not typically encountered in mental health contexts, influencing role boundaries, supervision needs and training requirements (Wobma et al., 2016; Levy et al., 2019; Wasilewski et al., 2023; Wasilewski et al., 2025). These findings indicate that neurorehabilitation peer support is not merely a direct extension of mental health models, but rather a contextually nuanced intervention that requires careful adaptation to the functional realities of brain injury.

A central tension identified in the data was the need to balance the value of living experiences with the requirement for the peer supporter to maintain professional standards. Wasilewski et al. (2025) recognized that professionals may find it challenging when sharing sensitive information for connecting individuals with lived experiences. Staff consistently described living experiences as offering unique empathy, relatability and emotional resonance, qualities that clinicians, despite their expertise, could not replicate. Yet concerns emerged regarding confidentiality, communication and role boundaries, particularly when cognitive, emotional or behavioural impairments were present following brain injury. These apprehensions resonate with previous findings in peer support research (Miyamoto & Sono, 2012), but are amplified in neurorehabilitation due to the variable effects of brain injury on insight, fatigue and executive functioning (Dillon et al., 2023; Jonasson et al., 2018). The data therefore underscore the importance of careful recruitment, reasonable adjustments and the development of tailored training and supervision frameworks that enable peer supporters to leverage their living experiences while reliably fulfilling professional responsibilities (Norton et al., 2025).

A critical contribution of this study is the identification of a gap in current training and implementation models. Staff highlighted that existing NHS peer support training, largely designed for mental health contexts, failed to address the cognitive, emotional and interpersonal challenges unique to brain injury. Many staff reported that this misalignment fostered misconceptions about the role and left teams ill‐equipped to support the peer supporter effectively. This gap points to the urgent need for a bespoke framework for neurorehabilitation peer support, incorporating modules on brain injury awareness, boundaries and safeguarding, communication strategies and structured supervision and reflective practice, essential to providing clarity, consistent support and sustainability in role delivery (Cooper et al., 2024; Magasi & Papadimitriou, 2022; Norton et al., 2025). Without such tailored infrastructure, peer support roles risk being misunderstood, underutilized or unsustainable in neurorehabilitation services.

The data also illustrated the transformative potential of the peer supporter to act as a bridge between clients, families and the clinical team. Staff highlighted that the role could enhance continuity of care, promote engagement in rehabilitation and provide a trusted source of hope and guidance, findings supported by peer‐mentoring programmes in traumatic brain injury showing enhanced coping, adaptation and quality of life for both clients and significant others (Hibbard et al., 2002). Peer‐led support groups in post‐stroke contexts have similarly demonstrated that experiential knowledge fosters meaningful social connection and supports functional participation (May et al., 2023). At the same time, participants raised important caveats: recovery narratives and role modelling may create unintended pressure if clients compare themselves unfavourably to the peer supporter's progress (Leamy et al., 2011). Staff emphasized the importance of managing expectations and providing structured guidance to ensure that inspiration offered by the peer supporter was balanced with realism. This was seen as essential to protecting both clients and peer supporters from potential distress or disengagement. Previous research highlights that unclear role boundaries and ambiguity can lead to emotional strain and burnout among peer workers, underscoring the need for well‐defined responsibilities and consistent supervision (Davidson et al., 2012; Gillard et al., 2014). Structured supervision approaches that promote ongoing clarification of role boundaries, facilitate reflective practice and offer tailored support have been found to mitigate these risks (Moran et al., 2013).

A key strength of this study is the depth of insight drawn from a highly experienced, interdisciplinary clinical team embedded in a real‐world neurorehabilitation service. The findings provide rich, contextually grounded perspectives on how peer support might function within the complexities of acquired brain injury care. However, several limitations must be acknowledged. Results are limited to staff perceptions from a single service and further research is needed to explore the perspectives of clients, families and staff across multiple sites. The small sample size (*n* = 8), although appropriate for an in‐depth qualitative exploration, limits the breadth of perspectives captured. Additionally, one focus group comprised only two participants, resulting in a dyadic interaction rather than a traditional group discussion. While dyads can generate meaningful and productive dialogue in qualitative research (Szulc & King, 2022), this may have shaped the dynamics and depth of discussion across data sources. Nonetheless, using a consistent topic guide and analytic framework ensured comparability and coherence across the dataset. Additionally, the sample comprised only female participants and was weighted towards occupational therapists, which may have influenced how relational dynamics, communication styles and support roles were perceived. Gendered and discipline‐specific experiences can shape understandings of collaborative practices and care delivery within rehabilitation teams (Martinez et al., 2012). Future studies should therefore aim to recruit more diverse samples, including a broader range of professions, genders and service contexts, to explore whether and how these factors influence attitudes towards peer support implementation.

## CONCLUSION

Overall, the findings support the development of a co‐produced, neuro‐specific framework for implementing peer support in rehabilitation services. Future research should involve individuals with living experience, clinicians, educators and service leaders in the co‐design of training, supervision and integration strategies. Pilot evaluations should assess the impact of peer supporter integration on client outcomes, family experiences and team functioning.

In conclusion, this study highlights both the promise and complexity of introducing a peer support worker role into neurorehabilitation. Staff viewed the role as deeply meaningful and potentially transformative, but also requiring thoughtful adaptation to the unique challenges of brain injury. Existing models, primarily derived from mental health paradigms, fall short in preparing both peer supporters and teams for these realities. To fully realize the benefits of peer support in neurorehabilitation, a tailored, context‐specific framework is urgently needed; one that centres on living experience while safeguarding role clarity, ensuring appropriate training and supporting sustainable integration within multidisciplinary services.

## AUTHOR CONTRIBUTIONS

**Stephen Dunne:** Conceptualization; investigation; writing – original draft; methodology; writing – review and editing; formal analysis; project administration. **Adele Simpson:** Conceptualization; methodology; writing – review and editing; formal analysis. **Jaden Allan:** Investigation; writing – review and editing; writing – original draft; formal analysis.

## Data Availability

The current article is accompanied by the relevant raw data generated during and/or analysed during the study, including files detailing the analyses and either the complete database or other relevant raw data. These files are openly available in the Open Science Framework at https://osf.io/xr6vm.
